# Preparation and Characterization of Solid Acid Catalysts for the Conversion of Sesamin into Asarinin in Sesame Oil

**DOI:** 10.3390/foods11091225

**Published:** 2022-04-24

**Authors:** Qiong Yu, Xue-De Wang, Hua-Min Liu, Yu-Xiang Ma

**Affiliations:** Institute of Special Oilseed Processing and Technology, College of Food Science and Engineering, Henan University of Technology, Zhengzhou 450001, China; zqymmh@163.com (Q.Y.); liuhuamin5108@163.com (H.-M.L.); myx366@163.com (Y.-X.M.)

**Keywords:** asarinin, solid acid catalyst, characterization, optimization, physicochemical properties

## Abstract

Asarinin, an isomer of sesamin, has attracted attention because it has stronger biological properties than sesamin. The research on the conversion of sesamin into asarinin is limited. In this study, solid acid catalysts were screened and applied to promote the conversion of sesamin into asarinin in sesame oil. The results showed that citric acid loaded on zeolite beta (CTAH) was the optimal catalyst for asarinin production among the prepared catalysts. Characterization showed that CTAH had the greatest pore volume, largest surface area and strongest acid content. Response surface methodology (RSM) was applied to optimize the reaction conditions for asarinin yield using CTAH. The optimal reaction conditions were as follows: temperature, 85 °C; time, 2.7 h; catalyst amount, 1.6%. The predicted and experimental values of asarinin yield were 50.79 and 51.80 mg/100 g, respectively. The peroxide value and color in sesame oil samples treated with CTAH were clearly improved. In short, CTAH is a solid acid catalyst with potential application in the industrial conversion of sesamin into asarinin and in the improvement of sesame oil.

## 1. Introduction

There is growing interest in lignans because they have many important physiological properties, including anti-inflammatory, antineoplastic and hypocholesterolemic effects [1,2,3]. Among the various lignans found in *Sesamum indicum* L., asarinin, an isomer of sesamin and a fat-soluble furanofuran-type lignan, has particularly attracted attention due to its plentiful biological properties [4,5,6]. Asarinin has been shown to reduce basal and TNF-α-induced vascular smooth muscle cell migration and proliferation, thus preventing atherosclerosis [5]. It has been reported that asarinin significantly inhibits the decrease in SOD activity caused by 6-hydroxydopamine in PC12 cells [7]. Asarinin also has been shown to inhibit mast cell activation by preventing the phosphorylation of Src family kinases [6].

Asarinin has stronger biological activity than sesamin, particularly in promoting fatty acid oxidizing liver enzyme gene expression and decreasing cholesterol. The hepatic concentrations of phospholipid were significantly higher in rats fed asarinin (42.2 ± 0.9 μmol/g) than in animals fed sesamin (37.9 ± 1.2 μmol/g) [8]. Kuo et al. found that, at the concentration of 32.25 μM, asarinin had a superoxide anion scavenging activity rate of 20.4%, whereas the scavenging activity rate of sesamin was 12.1% [9]. It was reported that asarinin showed remarkably stronger antioxidant activity in DPPH (IC_50_ = 0.21 mg/mL) radical scavenging activities than sesamin (IC_50_ = 3.05 × 10^10^ mg/mL) [10]. During the acid bleaching and deodorization of sesame oil processing, one of the major lignans, sesamin, is converted to asarinin [11,12]. The conversion of sesamin to asarinin is reversible; the ratio of sesamin and asarinin remains more or less the same as the reaction proceeds [11,13,14].

**Figure 1:**
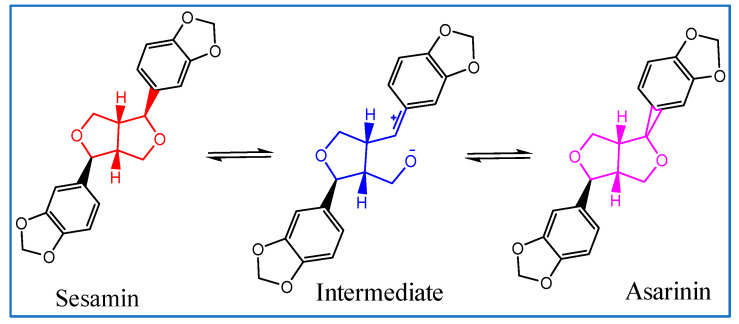
The mechanism of conversion of sesamin into asarinin.

The mechanism of conversion of sesamin into asarinin is displayed in Figure 1 [11,13,14,15]. Different catalysts have been used for the conversion of sesamin to asarinin. Tsai et al. reported that no asarinin was observed, regardless of the ratio, in a mixture of sesamin and sesamolin treated with sulfuric acid or formic acid in anhydrous toluene [11]. Wang et al. reported that the highest percentage of asarinin (53.0%) was achieved using hydrochloric acid as a catalyst in an ethanol–sesamin system. They also demonstrated that, in an ethanol–sesamin system, nearly 60.0% asarinin was produced when hydrochloric acid and ferric chloride were added in a certain proportion [16]. The content of asarinin reached 2.06 μg/mL when sesame samples were treated with hydrochloric acid [14]. Although these homogenous catalysts exhibit good activity, they have several limitations, such as the difficulty of catalyst removal and corrosion [17]. In contrast, solid acid catalysts have remarkable advantages including reusability, separability, and efficiency in the conversion of sesamin to asarinin [18]. Zeolite is a widely reported solid acid catalyst. Zeolite beta, having high acidity and pore structures, exhibits a high catalytic activity among various zeolites. Many recent studies have reported using modified zeolite for acid-catalyzed reactions such as esterification of free fatty acids and isomerization of alkanes [18,19].

At present, the research on the solid acid-catalyzed conversion of sesamin into asarinin is limited. As a result, this study represents exploration of a new catalyst which may be expected to be active in converting sesamin to asarinin in cold-pressed sesame oil, thereby enhancing the nutritional value of sesame oil. The best of the catalysts, namely citric acid loaded on zeolite beta (CTAH), was then investigated in depth. The parameters of temperature, time, catalyst amount and the concentration of citric acid loaded on zeolite beta were evaluated, and their effects on asarinin yield and the physicochemical properties of sesame oil were also assessed.

## 2. Materials and Methods

### 2.1. Materials and Chemicals

Sesamin with a purity exceeding 98.0% was purchased from Shanghai Macklin Biochemical Co., Ltd. (Shanghai, China). Asarinin with a purity exceeding 98.0% was obtained from Solarbio^®^ Life Sciences Co., Ltd. (Beijing, China). All standards were stored at 4 °C in darkness. The hydrogen type of zeolite beta (Hβ) with an Si/Al ratio of 25 was obtained from the Nankai University catalyst plant (Tianjin, China). Citric acid (CTA) was obtained from Shanghai Yuanye Biological Technology Co., Ltd. (Shanghai, China). The analytical grade chemicals including phosphotungstic acid (PTA), phosphomolybdic acid (PMA), ferric sulfate (FCS), ferrous sulfate (FRS), and ferric chloride hexahydrate (FCH) were obtained from Tianjin Kermel Chemical Reagents Co., Ltd. (Tianjin, China). Cold-pressed sesame oil was produced in a hydraulic press (Bafang Ltd., model XL-600, Suzhou, China).

### 2.2. Synthesis and Characterizaiton of Catalysts

#### 2.2.1. Catalyst Synthesis

Various solid acid catalysts were synthesized according to the impregnation method using Hβ as carrier material; the prepared catalysts were denoted as FCSH, FRSH, FCHH, PMAH, PTAH, and CTAH [20,21]. The impregnation procedure was carried out by soaking about 10.0 g of Hβ in 100 mL 0.1 mol/L reagent solution and continuously stirring at room temperature for 16.0 h. After the impregnation process was finished, the mixture was centrifuged. The samples were then dried, except CTA, which had to be first washed with distilled water to neutral and then dried. All the solids were dried at 65 °C, then calcined at 500 °C for 4.0 h. BHβ was prepared by calcinating Hβ directly in a muffle furnace. DHβ was obtained by modifying Hβ with 0.1 mol/L NaOH based on the literature [22]. DBHβ was prepared by modifying BHβ in the same way [19]. The prepared catalysts together with their abbreviations are shown in Table 1.

#### 2.2.2. Catalyst Characterization

The Brunauer–Emmett–Teller (BET) surface area measurements of various catalysts were performed using a 3-Flex automatic specific surface area analyzer (Micromeritics, USA) after removing N_2_. The pore size and pore volume were calculated by the BET method using experimental points, and pore size was calculated by Barrett–Joyner–Halenda (BJH) analysis [23]. NH_3_-TPD profiles (NH_3_ temperature programmed desorption) of different catalysts samples were recorded on a chemisorption analyzer (Xian Quan, TP-5080, Tianjin, China) [24]. Samples were pre-treated at 500 °C with a heating ramp: 10 °C/min by passing high purity helium for 1 h, and then cooled to 120 °C. Then, purity anhydrous ammonia was introduced to the saturation of the sample and the TPD analysis was performed from room temperature to 600 °C at a heating rate of 10 °C/min.

### 2.3. Effect of Catalyst on Asarinin Yield

Prior to response surface methodology (RSM), a serious of experiments were used to select the ranges for independent variables. Various reaction temperatures (30, 40, 60, 80, 100, 120 and 140 °C) were used when reaction time, catalyst amount (w_catalyst_/w_oil_) and the concentration of citric acid loaded on Hβ were fixed at 2.0 h, 1.0% and 0.4 mol/L, respectively. Reaction times of 0.5, 1.0, 1.5, 2.0, 2.5, 3.0 and 4.0 h were tested, when reaction temperature, catalyst amount (w_catalyst_/w_oil_) and the concentration of citric acid loaded on Hβ were fixed at 80 °C, 1.0% and 0.4 mol/L, respectively. As for catalyst amount (w_catalyst_/w_oil_), 0.2, 0.4, 0.6, 0.8, 1.0, 1.2, 1.4 and 1.6% were used when reaction time, temperature and the concentration of citric acid loaded on Hβ were fixed at 2.0 h, 80 °C and 0.4 mol/L, respectively. Finally, the concentrations of citric acid loaded on Hβ of 0.1, 0.2, 0.4, 0.6, 0.8, 1.0 and 1.5 mol/L were used when reaction time, temperature and catalyst amount (w_catalyst_/w_oil_) were fixed at 2.0 h, 80 °C and 1.0%, respectively.

The statistical analysis and optimization of data were performed by the Design-expert 8.0 statistical software package [25,26,27]. The optimal reaction conditions of asarinin yield were obtained through the Box–Behnken design.

### 2.4. Preparation of the Tested Sesame Oil Samples

All the tested sesame oil samples were prepared under the optimal reaction conditions as indicated by RSM. The oil samples were cooled at room temperature naturally, and then centrifuged (4000 r/min, 5 min), filtered and stored at −20 °C. Cold-pressed sesame oil without any treatment was recorded as CSO, and cold-pressed sesame oil samples treated with citric acid and with citric acid loaded on Hβ were denoted as sesame oil sample CTA and sesame oil sample CTAH, respectively.

### 2.5. Effect of Catalyst on the Physicochemical Properties of Sesame Oil

#### 2.5.1. Acid Value (AV), Peroxide Value (POV) and Color

Peroxide value and acid value were measured following previously reported methods [28]. The color of sesame oil was detected by a PFX-I series spectro-colorimeter (Lovibond Co., Ltd., Amesbury, UK). The optical path was 25.4 mm.

#### 2.5.2. Determination of Lignans

**Figure 2:**
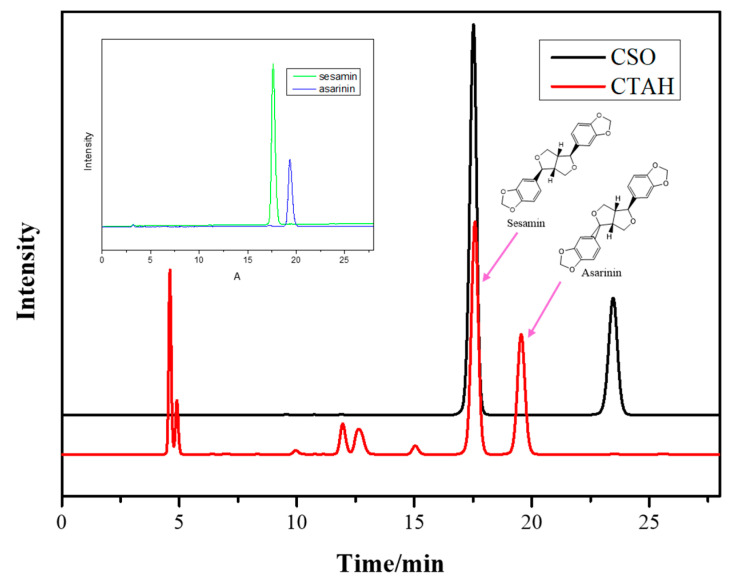
HPLC-UV chromatogram of the standard substance (A); cold-pressed sesame oil (CSO) and CSO were treated with citric acid loaded on Hβ (CTAH). Detection wavelength, 287 nm. Retention times and chemical structures of sesamin and asarinin in sesame oil are displayed.

Lignans were measured based on the method published in the literature [14,29]. The determinations of sesamin and asarinin were performed using the high-performance liquid chromatography equipped with ultraviolet-visible detector (HPLC-UV) method; HPLC-UV chromatograms of cold-pressed sesame oil (CSO) and CSO treated with citric acid loaded on Hβ (CTAH) are displayed in Figure 2.

### 2.6. Statistical Analysis

All measurements were conducted in triplicate. Probability values were considered statistically significant with one-way ANOVA using the SPSS package version 19.0. All significant differences were assessed by Duncan’s test. Values are denoted as means ± standard deviations.

## 3. Results and Discussion

### 3.1. Catalyst Screening

**Figure 3:**
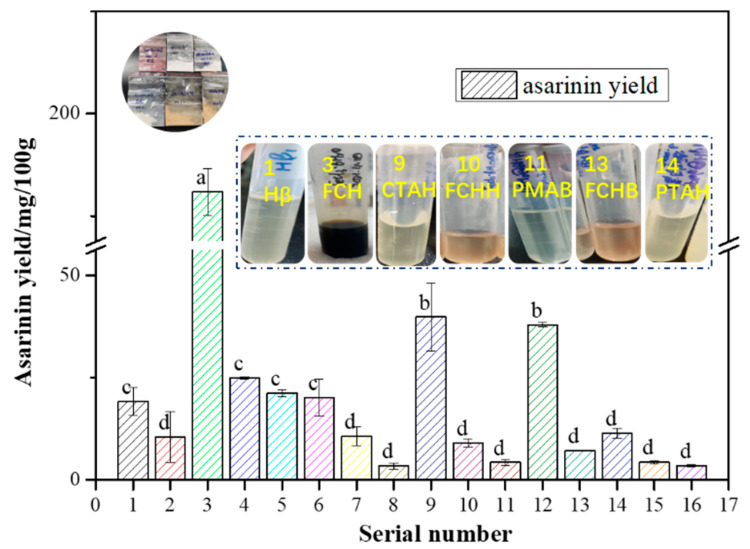
Effects of various catalysts on the yield of asarinin under the following reaction conditions: reaction temperature, 80 °C; reaction time, 2 h; catalyst amount, 1.0%. Different letters above the bars show significant differences among various treatments. The various solid acid catalysts represented by the serial number in the Figure are shown in Table 1.

The sample names of the various types of catalysts prepared are shown in Table 1. The effects of different types of catalysts on the conversion of sesamin into asarinin are displayed in Figure 3. The reaction conditions were as follows: temperature, 80 °C; time, 2 h; catalyst amount, 1.0%. As illustrated in Figure 3, there was no significant difference among the sesame oils treated with Hβ (No. 1 in Figure 3), DHβ (No. 5 in Figure 3) and BHβ (No. 6 in Figure 3), indicating that the modification of Hβ had no effect on the production of asarinin. Compared with the pure reagents PMA (No. 2 in Figure 3) and PTA (No. 4 in Figure 3), the influences of PMAH (No. 8 in Figure 3) and PTAH (No. 14 in Figure 3) on the conversion of sesamin in CSO were significantly reduced. Asarinin was not detected in the sesame oil samples treated with FRS and FCS, which are not shown in this figure. Figure 1 shows that the maximum amount of asarinin was produced when the sesame oil sample treated with FCH (No. 3 in Figure 3), and the next was the sesame oil sample treated with CTAH (No. 9 in Figure 3), demonstrating that FCH and CTAH could catalyze the production of asarinin. Apparently, in the CSO samples treated with FCSH (No. 16 in Figure 3), FCHH (No. 10 in Figure 3), FRSH (No. 15 in Figure 3) and PMAH (No. 8 in Figure 3), very little asarinin was produced at the reaction temperature of 80 °C. The reason for this may be that the active sites of the Hβ were occupied by these chemical regents [30,31]. In other words, the catalytic effect could not be enhanced with FCH, PMA or PTA loaded on Hβ. The color of the sesame oil sample treated with FCH was dark brown. Although this sample showed excellent catalysis, this color of sesame oil in food products would not be accepted by consumers. Thus, CTAH was chosen as the best catalyst for further study.

### 3.2. Catalyst Characterization

#### 3.2.1. Nitrogen Adsorption and Desorption Analysis

**Figure 4:**
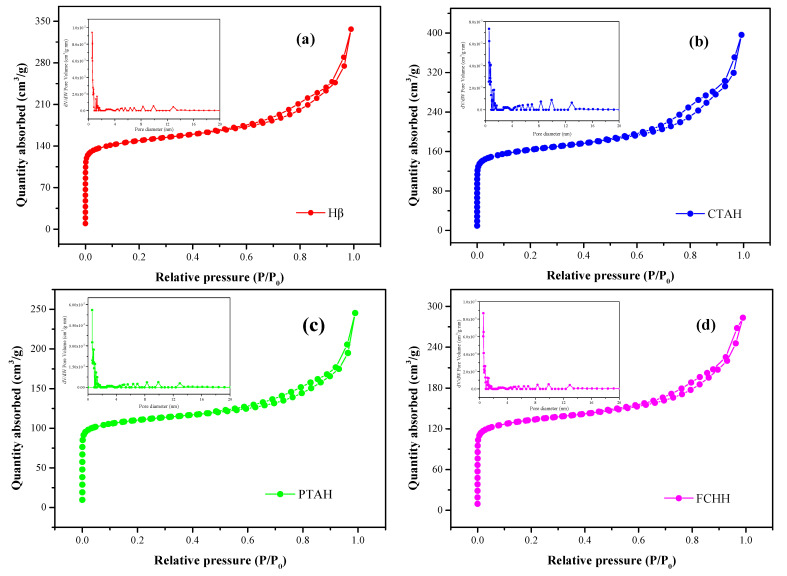

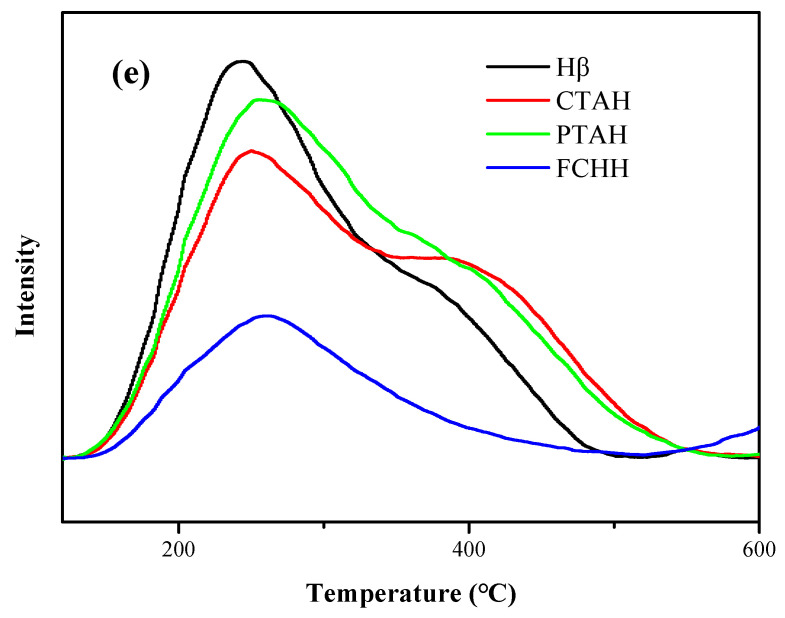
(**a**–**d**): N_2_ adsorption and desorption isotherms and pore size distribution of various catalysts; (**e**) NH_3_-TPD curves of various catalysts. Hβ: Hydrogen type of zeolite beta; CTAH: Citric acid loaded on Hβ; PTAH: Phosphotungstic acid loaded on Hβ; FCHH: Ferric chloride hexahydrate loaded on Hβ.

The structure properties including surface area, pore size and pore volume of different catalysts were obtained from nitrogen (N_2_) adsorption and desorption isotherms (Figure 4a–d and Table 2). The catalysts Hβ, CTAH, PTAH and FCHH exhibited a combination of type I and type IV isotherms, on the basis of the classification of international union of pure and applied chemistry (IUPAC). It is clear that, at lower relative pressures (P/P_0_ = 0.0–0.1), the catalysts exhibited isotherm type I, which corresponds to microporous material. A hysteresis loop was observed at the upper section of isotherm over the relative pressure range from 0.6 to 0.9, indicating that there were mesopores in the solid. This is further confirmed in the BET analysis results in Table 2. The surface area, pore size, micropore volume and mesopore volume of CTAH were the biggest among the selected catalysts because the solid contains both micropores and mesopores. Similar phenomena have been reported previously [32,33].

#### 3.2.2. Acidic Properties

Figure 4e shows that two NH_3_ desorption peaks are present in the NH_3_-TPD profiles of Hβ, CTAH, PTAH and FCHH catalysts. According to the literature [19,34], the strong peaks in the 150–300 °C range are assigned to the weak acid sites in the catalysts, and peaks in the 300–500 °C range can be ascribed to the strong acid sites. The acid amounts and strengths of different catalysts are displayed in Table 2. The number of acid sites of the samples was determined by the amount of desorbed ammonia, and acidic strength was determined by desorption temperature [21,34]. Compared with Hβ, the number of weak acid sites of CTAH, PTAH and FCHH was less by 19.43%, 4.27% and 56.87%, respectively; the number of strong acid sites of CTAH and PTAH was greater by 67.21% and 60.66%, respectively. The order of the ratio of strong/weak was: CTAH (0.60) > PTAH (0.49) > Hβ (0.29) > FCHH (0.04). The results indicate that CTA and PTA modification favors the formation of strong acid sites [20,21]. In general, the acid strength of CTAH was strongest among the prepared catalysts.

### 3.3. Effect of Catalyst on Asarinin Yield

According to the characterization and these preliminary tests of various solid acid catalysts, CTAH showed the best compromise between activity and structural properties; it had: (1) the highest pore volume (0.61 cm^3^/g) and surface area (537.99 m^2^/g); (2) the highest strong acidity (0.10 mmol/g); and (3) high asarinin yield (39.79 mg/100 g). The reason for the good catalytic effect may be that the change in catalyst structure makes it more suitable for the conversion of sesamin into asarinin. Consequently, the effects of CTAH on the reaction conditions for asarinin yield were further investigated.

#### 3.3.1. Single Factors Analysis

**Figure 5:**
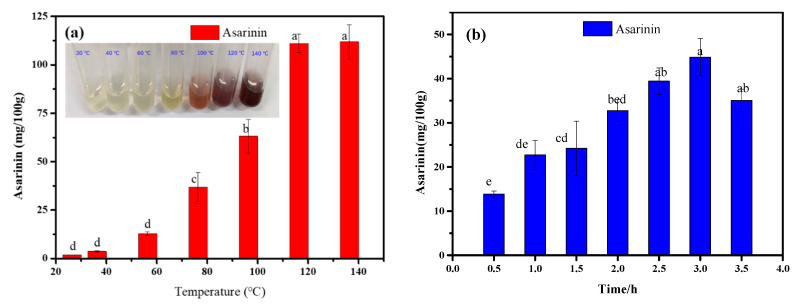

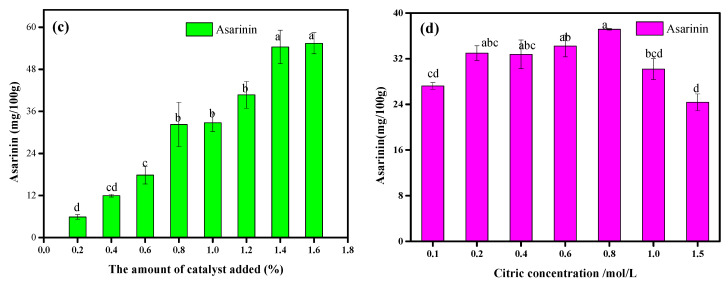
Effects of various reaction conditions on asarinin production: (**a**) catalyst amount, 1.0%; reaction time, 2.0 h; concentration of citric acid loaded on Hβ, 0.4 mol/L; (**b**) catalyst amount, 1.0%; reaction temperature, 80 °C; concentration of citric acid loaded on Hβ, 0.4 mol/L; (**c**) reaction temperature, 80 °C; reaction time, 2.0 h; concentration of citric acid loaded on Hβ, 0.4 mol/L; (**d**) catalyst amount, 1.0%; reaction temperature, 80 °C; reaction time, 2.0 h. Different letters above the bars show significant differences among various conditions.

The effects of reaction temperature, time, catalyst amount and the concentration of CTA loaded on Hβ on asarinin production are shown in Figure 5a–d. It can be seen that the content of asarinin increased gradually with increases in time, temperature and catalyst amount. Different concentrations of CTA loaded on Hβ had little effect on asarinin yield.

As shown in Figure 5a, the yield of asarinin increased within the reaction temperature range from 30 to 140 °C. In terms of the sensory qualities of sesame oil, flavor and color underwent significant deterioration at the temperatures above 100 °C (see the picture in Figure 5a). Therefore, 80 °C was selected as the appropriate temperature for further study. The production of asarinin increased with reaction time from 0.5 to 3.0 h, and then decreased after 3.0 h (Figure 5b). The yields of asarinin were 39.44 and 44.87 mg/100 g at 2.5 and 3.0 h, respectively, and there was no significant difference between them. Considering the cost of time and energy, 2.5 h was chosen as the time for further tests. The variation in asarinin yield according to catalyst amount is displayed in Figure 5c. There was no significant difference in asarinin yield when catalyst amount varied between 1.4% and 1.6%. Considering the yield of asarinin, adding 1.6% catalyst was considered the better choice. The effects of different concentrations of CTA loaded on Hβ on the content of asarinin yield are displayed in Figure 4d. There was no significant difference in the production of asarinin when the concentration of citric acid was between 0.2 and 0.8 mol/L. As the concentration of citric acid increased, the yield of asarinin decreased. The reason for this was related to the activity of catalyst. According to the literature, high concentration of citric acid will lead to the collapse of the Hβ framework [20]. There was no significant difference in asarinin yield among different concentrations of citric acid loaded on Hβ (Figure 5d). Therefore, this factor was not taken into account in the following selection of response surface factor.

#### 3.3.2. Optimized Experiment

The yield of asarinin depends on reaction parameters such as reaction temperature, time and type of catalyst. The ranges of variables were decided on the basis of single factor experiments as reported above. In the present study, three factors (catalyst amount, reaction temperature and reaction time) were selected as the most significant in determining asarinin yield for RSM. These variables are presented in Table 3.

#### 3.3.3. Determination of Optimal Reaction Conditions

Table 4 exhibits the results of ANOVA analysis of asarinin yield. The model F-value of 16.97 and *p*-value of 0.0006 demonstrated statistical significance of the quadratic model. Coefficient of determination (R^2^) was 0.9562, which indicates that the model represented the data accurately. This demonstrated that the relationship between the independent variables (catalyst amount, temperature and time) and the response (asarinin yield) can be explained based on the regression model. Thus, this model was fit for the further analysis of the influence of process parameters.

**Figure 6:**
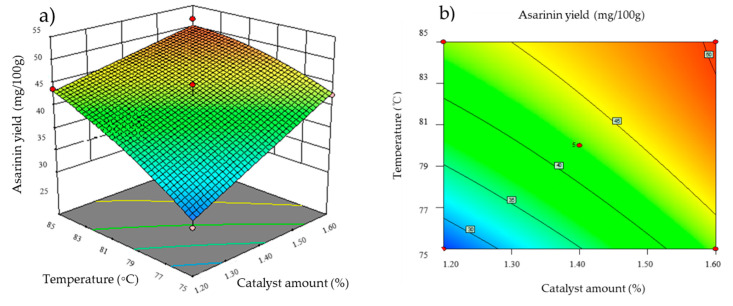

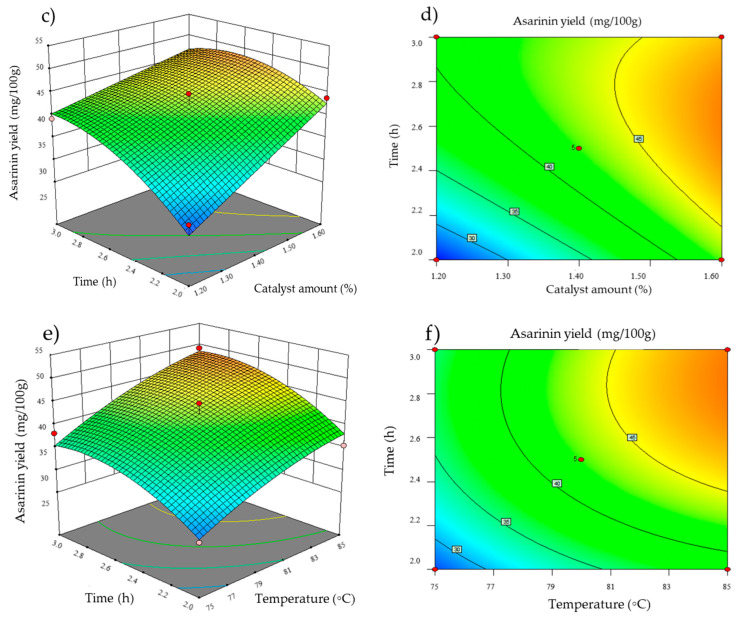
Response surface plots and corresponding contour plots of the effects of different factors on asarinin yield. Interaction of temperature and catalyst amount (**a**,**b**); interaction of time and catalyst amount (**c**,**d**); interaction of time and temperature (**e**,**f**).

The model equation was significant at 95% confidence level with non-significant lack of fit [25,27,35]. The goodness of fit of this model was verified by the coefficient of R^2^ (0.9456) and the adjusted R^2^ (0.8756). Nonsignificant lack-of-fit (0.06) in comparison with the pure error substantiated the fitness of the quadratic model. The empirical relationship between asarinin yield and the investigated variables in coded units is shown in the equation:

A = 42.39 + 5.81 X_1_ + 5.98 X_2_ + 4.68 X_3_ − 2.26 X_1_X_2_ − 2.60 X_1_X_3_ + 0.51 X_2_X_3_ − 0.02 X_1_^2^ − 1.58 X_2_^2^ − 3.50 X_3_^2^ (1)

where A is the yield of asarinin (response), and X_1_, X_2_, and X_3_ are the coded values of the experiment variables, namely catalyst amount (X_1_), temperature (X_2_) and time (X_3_). The influence of each factor was significant for the first-order linear effect (X_1_, X_2_, X_3_) (*p* < 0.05). All the quadratic terms X_1_^2^, X_2_^2^, X_3_^2^ and interactive effects (X_1_X_2_, X_1_X_3_, X_2_X_3_) were non-significant (*p* > 0.05). The results showed that the three main factors showed significant linear effects on the yield of asarinin. The 3D response surface and the response contour plots of the three independent variables are exhibited in Figure 6. The predicted asarinin yield was 50.79 mg/100 g under the optimal conditions: reaction temperature, 85 °C; time, 2.7 h; and catalyst amount, 1.6%. The experimental value was 51.72 mg/100 g.

### 3.4. Determination of Physicochemical Properties and Lignans of Tested Sesame Oils

Under the optimal reaction conditions, the effects of CTAH on the basic physicochemical properties of sesame oil samples were further evaluated. In this section, the acid value, peroxide value, color (red value, yellow value) and lignans of sesame oil samples treated with different methods were compared and analyzed. AV (acid value) and POV (peroxide value), the classic indicators applied to evaluate the oxidation degree of vegetable oils, were also detected here [36,37,38].

The acid values and peroxide values of the sesame oil samples CSO, CTA and CTAH are exhibited in Table 5. Acid value is a direct index of the percentage of free fatty acids in a certain amount of sesame oil. It is a measure of the degree to which the triglycerides in the oil have been decomposed into free fatty acids by lipase action or high temperature [37,39]. It can be seen from Table 5 that there was no significant difference among the tested sesame oil samples CSO, CTA and CTAH in terms of acid value. The peroxide value of sesame oil is commonly used as a quality parameter [37,40]. The peroxide values of the sesame oil samples are exhibited in Table 5. Obviously, the sesame oil sample treated with citric acid loaded on Hβ (CTAH) had the lowest peroxide value (0.14 meq/kg), followed by CSO (0.80 meq/kg). The peroxide values of the different treated sesame oil samples showed significant differences (*p* < 0.001). Compared with the treated sesame oil sample CSO, the peroxide value of the treated sesame oil sample CTAH was 82.5% less. This might be due to the lignans in the treated sesame oil samples [41].

Many published studies have demonstrated that sesamin can be converted into asarinin under the conditions of heat and acid [13,14,15]. The contents of sesamin and asarinin are exhibited in Table 5. There was no asarinin in the sesame oil samples CSO and CTA. In other words, CTA could not promote the conversion of sesamin. Compared with the sesame oil sample CTA, the sesamin content of sesame oil sample CTAH was significantly decreased by 27.8%, indicating that CTAH could markedly promote the production of asarinin. Compared with CSO, the red value and yellow value of the sesame oil sample CTAH were significantly reduced by 56.0% and 68.5%, respectively. The results showed that CTAH has a decolorization effect [42]. CTAH, an eco-friendly and sustainable solid acid catalyst, is expected to decolorize vegetable oils. The catalyst is used in the decolorization process in the refining of leaching sesame oil, which can not only have a decolorization effect, but also promote the conversion of sesamin into asarinin, thereby improving the added-value of leaching sesame oil.

## 4. Conclusions

In this study, various solid acid catalysts were prepared, characterized and screened according to their ability to convert sesamin into asarinin. The selected catalyst, citric acid loaded on zeolite beta (CTAH), was investigated for its ability to convert sesamin into asarinin. The effects of reaction conditions on asarinin yield and the physicochemical properties of sesame oil samples were assessed. The results showed that the catalyst CTAH was the best among the catalysts tested. CTAH had the greatest surface area, largest pores and strong acid sites. The optimal reaction conditions were: reaction temperature of 85 °C, reaction time of 2.7 h and catalyst amount of 1.6%. The predicted and experimental values of asarinin yield were 50.79 and 51.80 mg/100 g, respectively. Under the optimal reaction conditions, there was no significant difference among the acid values of sesame oil samples. Compared with the treated sesame oil sample CSO, the peroxide value of the treated sesame oil sample CTAH was 82.5% less. In summary, CTAH is a solid acid catalyst with great potential for industrial use in converting sesamin into asarinin, thereby not only improving the basic physicochemical properties of sesame oil, but also increasing its biological activity. This study provides a reference for the process conditions needed in using a solid acid catalyst to produce asarinin and improve cold-pressed sesame oil.

## Figures and Tables

**Table 1:** The various solid acid catalysts prepared in this study are listed in the table with their corresponding serial number and abbreviations.

Serial Number	Abbreviations	Sample Name
1	Hβ	Hydrogen type of zeolite beta
2	PMA	Phosphomolybdic acid
3	FCH	Ferric chloride hexahydrate
4	PTA	Phosphotungstic acid
5	DHβ	Alkali modified Hβ
6	BHβ	Calcined Hβ
7	DBHβ	Alkali modified BHβ
8	PMAH	Phosphomolybdic acid loaded on Hβ
9	CTAH	Citric acid loaded on Hβ
10	FCHH	Ferric chloride hexahydrate loaded on Hβ
11	PMAB	Phosphomolybdic acid loaded on BHβ
12	CTAB	Citric acid loaded on BHβ
13	FCHB	Ferric chloride hexahydrate loaded on BHβ
14	PTAH	Phosphotungstic acid loaded on Hβ
15	FRSH	Ferrous sulfate loaded on Hβ
16	FCSH	Ferric sulfate loaded on Hβ

**Table 2:** Surface and acidic properties of various solid acid catalysts.

		Hβ	CTAH	PTAH	FCHH
Surface area (m^2^/g)		489.39	537.99	360.61	435.70
External surface area (m^2^/g)		134.90	178.27	96.76	129.29
Pore size (nm)		4.27	4.57	4.22	4.04
Pore volume (cm^3^/g)		0.52	0.61	0.38	0.44
Micropore volume (cm^3^/g)		0.17	0.18	0.13	0.15
Mesopore volume (cm^3^/g)		0.35	0.44	0.25	0.29
NH_3_ acidity (mmol/g)	weak	0.21	0.17	0.20	0.09
	strong	0.06	0.10	0.10	0.00
	strong/weak	0.29	0.60	0.49	0.04
Acid strength (°C)	weak	244	251	256	261
	strong	351	351	351	600

Notes: Hβ: Hydrogen type of zeolite beta; CTAH: Citric acid loaded on Hβ; PTAH: Phosphotungstic acid loaded on Hβ; FCHH: Ferric chloride hexahydrate loaded on Hβ.

**Table 3:** The levels of independent variables used for RSM.

Level/Variables	Catalyst Amount (X_1_)/%	Reaction Temperature (X_2_)/°C	Reaction Time (X_3_)/h
−1	1.2	75	2.0
0	1.4	80	2.5
1	1.6	85	3.0

**Table 4:** ANOVA analysis on asarinin yield.

Source	Sum of Squares	Degree of Freedom	Mean Square	F Value	*p*-Value Prob > F	
Model	844.84	9	93.87	16.97	0.0006 *	significant
X_1_	270.12	1	270.12	48.84	0.0002 *	
X_2_	285.78	1	285.78	51.67	0.0002 *	
X_3_	175.17	1	175.17	31.67	0.0008 *	
X_1_X_2_	20.46	1	20.46	3.70	0.0959	
X_1_X_3_	27.01	1	27.01	4.88	0.0628	
X_2_X_3_	1.04	1	1.04	0.19	0.6775	
X_1_^2^	0.00	1	0.00	0.00	0.9870	
X_2_^2^	10.56	1	10.56	1.91	0.2095	
X_3_^2^	51.67	1	51.67	9.34	0.0184	
Residual	38.72	7	5.53			
Lack of Fit	31.50	3	10.50	5.82	0.0610	not significant
Pure Error	7.22	4	1.80			
Cor Total	883.56	16				
Std. Dev.	2.35		R-Squared	0.9562		
Mean	39.99		Adj R-Squared	0.8998		
C.V.%	5.88		Pred R-Squared	0.4169		
Press	515.23		Adeq Precision	13.6019		

* Values are significant at 95% confidence level.

**Table 5:** Acid value, peroxide value and lignans of different treated sesame oil samples.

Samples	Acid Value (mg KOH/g)	Peroxide Value (meq/kg)	Sesamin (mg/100 g)	Asarinin (mg/100 g)	Red Value	Yellow Value
CSO	1.95 ± 0.05 ^a^	0.80 ± 0.02 ^b^	489.74 ± 2.89 ^a^	Nd	1.25 ± 0.07 ^a^	4.60 ± 0.14 ^a^
CTA	2.06 ± 0.16 ^a^	1.82 ± 0.03 ^a^	497.20 ± 2.24 ^b^	Nd	0.90 ± 0.14 ^b^	3.75 ± 0.21 ^b^
CTAH	1.92 ± 0.00 ^a^	0.14 ± 0.00 ^c^	358.99 ± 0.83 ^c^	83.52 ± 0.52 ^a^	0.55 ± 0.07 ^c^	1.45 ± 0.07 ^c^

Notes: Values are means ± SD (standard deviation), mean value of different superscript letters in the same column are significantly different at *p* < 0.05. nd represents not detected. CSO: cold-pressed sesame oil; CTA: CSO treated with citric acid; CTAH: CSO treated with citric acid loaded on Hβ.

## Data Availability

The data presented in this study are available in this article.
